# TiO_2_ Nanostructures for Photoelectrocatalytic Degradation of Acetaminophen

**DOI:** 10.3390/nano9040583

**Published:** 2019-04-09

**Authors:** Joan Borràs-Ferrís, Rita Sánchez-Tovar, Encarnación Blasco-Tamarit, Maria José Muñoz-Portero, Ramón M. Fernández-Domene, José García-Antón

**Affiliations:** Ingeniería Electroquímica y Corrosión (IEC), Departamento de Ingeniería Química y Nuclear, ETSI Industriales, Universitat Politècnica de València, Camino de Vera s/n, 46022 Valencia, Spain; jborras.93@gmail.com (J.B.-F.); meblasco@iqn.upv.es (E.B.-T.); mjmunoz@iqn.upv.es (M.J.M.-P.); raferdo1@etsii.upv.es (R.M.F.-D.)

**Keywords:** acetaminophen, photoelectrodegradation, pH, nanostructures, titanium dioxide, anodization

## Abstract

Advanced oxidation processes driven by renewable energy sources are gaining attention in degrading organic pollutants in waste waters in an efficient and sustainable way. The present work is focused on a study of TiO_2_ nanotubes as photocatalysts for photoelectrocatalytic (PEC) degradation of acetaminophen (AMP) at different pH (3, 7, and 9). In particular, different TiO_2_ photocatalysts were synthetized by stirring the electrode at different Reynolds numbers (Res) during electrochemical anodization. The morphology of the photocatalysts and their crystalline structure were evaluated by field emission scanning electron microscopy (FESEM) and Raman confocal laser microscopy (RCLM). These analyses revealed that anatase TiO_2_ nanotubes were obtained after anodization. In addition, photocurrent densities versus potential curves were performed in order to characterize the electrochemical properties of the photocatalysts. These results showed that increasing the Re during anodization led to an enhancement in the obtained photocurrents, since under hydrodynamic conditions part of the initiation layer formed over the tubes was removed. PEC degradation of acetaminophen was followed by ultraviolet-visible absorbance measurements and chemical oxygen demand tests. As drug mineralization was the most important issue, total organic carbon measurements were also carried out. The statistical significance analysis established that acetaminophen PEC degradation improved as hydrodynamic conditions linearly increased in the studied range (*Re* from 0 to 600). Additionally, acetaminophen conversion had a quadratic behavior with respect to the reaction pH, where the maximum conversion value was reached at pH 3. However, in this case, the diversity of the byproducts increased due to a different PEC degradation mechanism.

## 1. Introduction

In recent years, several studies have shown interest in the degradation of pharmaceuticals, which are considered to be emerging pollutants due to the fact that they remain unregulated or are currently undergoing a regularization process [1]. However, these emerging pollutants are continuously introduced into the environment and can affect water quality even in small concentrations [1]. These compounds can be incorporated into waterbodies from different sources, such as excretion by humans and animals [2] and the pharmaceutical industry [3].

In particular, acetaminophen (AMP), also known as paracetamol (*N*-acetyl-4-aminophenol, Figure S1), is a common analgesic and antipyretic drug that is widely used all over the world. AMP has attracted the interest of researchers due to its tendency to induce liver and kidney damage. Besides, there have been several reports that have supported the presence of AMP in rivers, ambient waterways, and the influents and effluents of wastewater treatment plants [4]. 

Additionally, several pharmaceuticals, such as AMP, are neither removed during wastewater treatment nor biodegraded in the environment [5] due to their stable chemical structure. For that reason, many researchers have focused their attention on advanced oxidation processes (AOPs), where a strongly oxidizing hydroxyl radical (OH) is created in situ: Therefore, this radical could oxidize a wide range of organic pollutants present in the water until their degradation [6].

Recently, different AOPs have been used in order to remove AMP from waterbodies, including ozonation and H_2_O_2_/UV oxidation methods [2]. However, heterogenous photocatalysis and photoelectrocatalysis are two of the most promising AOPs for the destruction of aquatic pollutants, such as AMP. Heterogenous photocatalysis is a technology based on a semiconductor as a photocatalyst, which should be able to transform the energy from electromagnetic radiation into chemical energy (i.e., the semiconductor takes advantage of electromagnetic radiation to generate a hydroxyl radical). Moreover, with this method, it is possible to achieve the degradation and complete mineralization of environmental pollutants [7]. Nevertheless, the efficiency of heterogenous photocatalysis is very low, since organic compounds are slowly degraded [3], and for that reason, an external anodic potential can be applied over the semiconductor in order to reduce the recombination of photogenerated electron/hole pairs [8].

Apart from this, to enhance the degradation rate in heterogenous photoelectrocatalysis, it is crucial to design efficient photocatalysts. TiO_2_, mainly in its anatase phase, has been the most widely used photocatalyst due to its ability to oxidize dissolved organic compounds and its nontoxicity, low cost, and long-term photostability [3]. For this application, it is essential to maximize the specific surface area in order to obtain a high overall efficiency (due to, for example, the increase in the surface to photogenerate electron and hole pairs [9]), and thus TiO_2_ in its nanoparticulated form is widely used. In addition to this, TiO_2_ nanotubes might allow for a much higher control of chemical or physical behavior. In this way, when the photocatalyst dimensions to the nanoscale decrease, apart from increasing the specific area, the electronic properties may also change, and consequently kinetics may be enhanced [10,11,12,13]. On the other hand, TiO_2_ can be synthesized in the form of nanotube arrays in order to achieve a high surface area and consequently enhance photocatalytic activity [10]. Among all the methods used to synthesize nanotubes, the anodization of titanium in fluoride-based electrolytes is one of the most promising due to its ability to obtain highly ordered nanotube arrays and its control over their dimensions [14]. 

The main drawback of anodization in organic electrolytes is the formation of an initiation layer over the tubes, which decreases the efficiency of the photocatalytic process, and consequently the efficiency of AMP is reduced. For that reason, the use of hydrodynamic conditions in the anodization procedure with a rotating disk electrode configuration is gaining interest in order to remove the “initiation layer” during the anodization process [15].

On the other hand, several studies have been focused on increasing the efficiency of the AOPs in AMP degradation by changing different operation parameters, such as the pH. However, the influence of the pH is still not clear, since according to the literature, either acid [16] or alkaline pH [2,6] is found to be the optimum for the photodegradation of acetaminophen. Therefore, the present study tried to elucidate these differences based on the degradation mechanism of AMP.

Accordingly, the main goal of this work was to evaluate pH influence as an operation parameter during AMP photoelectrocatalytic (PEC) degradation with anatase TiO_2_ nanotubes anodized under hydrodynamic conditions. In addition, a statistical study was carried out in order to study the influence of pH and hydrodynamic conditions, as well as the interaction between them. To characterize the nanostructures, different microscopy techniques were used: Field emission scanning electron microscopy (FE-SEM) and Raman confocal laser microscopy (RCLM). Additionally, the kinetic study was performed by using UV-visible spectroscopy, chemical oxygen demand (COD) tests, and total organic carbon (TOC) measurements.

## 2. Experimental Procedure

### 2.1. Preparation of the Photocatalyst

Titanium dioxide nanotubes were used as photocatalysts. In order to synthetize the nanostructures, titanium rods (8 mm in diameter and 99.3% purity) were anodized in ethylene glycol-based (99%, Panreac, Illinois Tool Works Inc. (ITW), Glenview, IL, USA) electrolytes containing 0.05 M NH_4_F (≥98%, Sigma-Aldrich, San Luis, MO, USA) and 1 M H_2_O. To prepare the rods, they were abraded with 220 to 4000 silicon carbide (SiC) papers (Struers), and then they were sonicated in ethanol for 2 min and dried with N_2_. Anodization was carried out under stagnant conditions (*Re* = 0) and by stirring the titanium rods using a rotating disk electrode (RDE, Metrohm Autolab, Utrecht, The Netherlands). This RDE was coupled to a motor controller with an accuracy of ±1 rpm in order to monitor the hydrodynamic conditions of the process. Figure S2a shows a scheme of the electrode configuration during anodization. The different Reynolds numbers used (0, 200, 400, and 600) corresponded to the following controlled rotation speeds: 0, 1728, 3456, and 5185 rpm, respectively. The equivalence of the rotation speed with Re was calculated as follows:

(1) Re=ω·r2·ρμ,

where ω is the rotation speed expressed in rad s^−1^, r is the radius of the working electrode in cm, and ρ and μ are the density in g cm^−3^ and dynamic viscosity in g cm^−1^ s^−1^ of the solution, respectively.

Anodization was performed in a two-electrode electrochemical cell at 55 V for 30 min and at room temperature. A titanium rod was the working electrode (with an exposed area of 0.5 cm^2^), and a platinum foil was the counter electrode. A multimeter (Tenma, OH, USA) was used in order to register the current density during anodization. After anodization, the samples were washed with ethanol and then dried with N_2_ (Figure S2b).

In order to transform amorphous TiO_2_ into an anatase phase structure, the anodized samples were annealed in a cylindrical oven (Carbolite, Sheffield, United Kingdom) at 450 °C for 1 h. 

### 2.2. Characterization of the Photocatalyst

#### 2.2.1. Morphological and Structural Characterization

For the morphological characterization of the nanostructures, a field emission scanning electron microscope (FE-SEM, Zeiss Ultra 55) was used. The crystalline structure was evaluated with a Raman confocal laser microscope (Witec, Ulm, Germany) provided by a 632-nm neon laser using 420 µW.

#### 2.2.2. Photoelectrochemical Characterization

For the photoelectrochemical characterization, a three-electrode cell configuration connected to an Autolab PGSTAT302N potentiostat was used under simulated sunlight conditions (LOT solar simulator), AM 1.5 (100 mW·cm^−2^). A 0.26-cm^2^ exposed area of the TiO_2_ nanotubes was used as a working electrode. An Ag/AgCl (3 M KCl) electrode was the reference electrode, and a platinum tip was the counter electrode (Figure S2b). 

Photocurrent versus potential characteristics were recorded by scanning the potential from −0.8 V_Ag/AgCl_ to 0.5 V_Ag/AgCl_ with a scan rate of 2 mV s^−1^ in 1 M KOH (85%, Panreac). Photocurrent transients as a function of the applied potential were recorded by chopped light irradiation (60 s in the dark and 20 s in the light).

### 2.3. Photoelectrocatalysis Experiments

The TiO_2_ nanotubes synthetized by electrochemical anodization were used for the PEC degradation of acetaminophen (≥99%, Sigma-Aldrich) under UV light at 360 nm, irradiating the photocatalyst perpendicularly (using a 1000-W Xenon Lamp (LOT) connected to a LOT monochromator and to an Autolab PGSTAT302N potentiostat with a photon beam diameter of 1 cm). The light power density was 40 mW·cm^−2^, i.e., a distance of 5 cm between the light source and the nanostructures. The geometric area of the TiO_2_ photocatalysts was 0.5 cm^2^, and the initial acetaminophen concentration was 40 mg·L^−1^. This concentration was selected because a previous concentration process before the PEC degradation might be recommended in order to treat paracetamol concentrations of the order of ppm. Three different pH levels were used (3, 7, and 9). A pH of 7 was the pH of the acetaminophen, and pH 3 was adjusted with HCl (37–38%, Panreac) and pH 9 with NaOH (98%, Panreac). The experiments were carried out under constant stirring and in a 40-mm Light Path Quartz suprasil cuvette (Hellma Analytics, Müllheim, Germany) containing 12 mL of AMP with the TiO_2_ photocatalyst as a working electrode, an Ag/AgCl 3-M KCl as a reference electrode, and a platinum tip as a counter electrode. For the PEC degradation tests, a potential of 0.5 V_Ag/AgCl_ was applied to the photocatalyst. Samples were equilibrated in the dark for 30 min before light exposure. In order to follow the removal of the acetaminophen, UV-visible spectrophotometer (Unicam, United Kingdom, UV/VID Spectrometer) measurements were carried out every 30 min until 4 h. Additionally, chemical oxygen demand tests were performed according to the “closed reflux, colorimetric method” described in Section 5220-D of the standard method: “5220 Chemical Oxygen Demand” [17]. Finally, a study of total organic carbon was carried out by means of a TOC-L Shimadzu analyzer. 

## 3. Results and Discussion

### 3.1. Photocatalyst Characterization 

**Figure 1:**
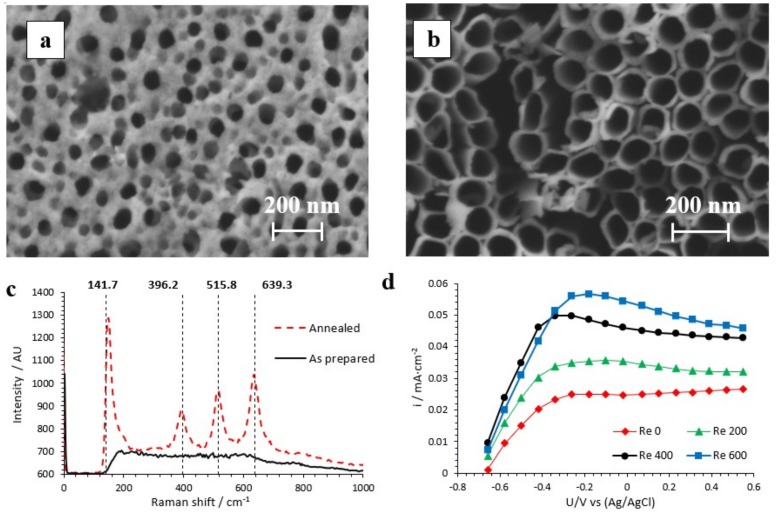
Field emission scanning electron microscopy (FE-SEM) images of the top view of the TiO_2_ nanotubes anodized at (**a**) *Re* = 0 and (**b**) *Re* = 600. (**c**) Raman confocal laser spectra of the as-prepared and annealed samples anodized at *Re* = 600 (at 450 °C for 1 h). (**d**) Maximum photocurrent densities versus potential curves of the samples anodized at *Re* = 0, 200, 400, and 600 (lengths = 3.7, 5.0, 5.4, and 6.1 µm, respectively) under AM 1.5 illumination.

Figure 1a,b shows the FE-SEM images of the photocatalysts obtained by electrochemical anodization of Ti. From Figure 1a, it is possible to discern that under static conditions, a porous TiO_2_ layer covered the nanotubes (i.e., an initiation layer): However, under hydrodynamic conditions this layer began to detach in certain areas (*Re* = 200 and 400) [15]. In those areas where the initiation layer was still attached, the diameter of the porosity of this layer increased with hydrodynamic conditions. Figure 1b shows that the initiation layer almost disappeared at *Re* = 600. The initiation layer blocked part of the solar radiation, which could not be absorbed by the semiconductor. Consequently, the PEC degradation efficiency, with nanotubes anodized without stirring the electrode, might have been reduced. On the other hand, when the initiation layer was not completely removed, an increase in the average diameter of the initiation layer porosity was observed as hydrodynamic conditions increased (at *Re* = 200 and 400) [15]. Thus, the entrance of the tubes became more accessible. The lengths of the nanotubes were 3.7 µm, 5.0 µm, 5.4 µm, and 6.1 µm when anodization was performed at *Re* = 0, 200, 400, and 600, respectively.

Raman confocal laser microscopy permitted an evaluation of the crystallinity of the photocatalysts. Figure 1c shows, as an example, the Raman spectra of an anodized (as prepared) and an annealed sample. For the annealed nanostructure, the four characteristic peaks of the anatase phase (141.7, 396.2, 515.8, and 639.3 cm^−1^) can be elucidated [18,19,20]. 

Figure 1d shows the maximum photocurrent densities as a function of the applied voltage under simulated sunlight conditions (100 mW·cm^−2^). In Figure 1d, it is important to highlight, first, that the higher the Reynolds number used during anodization, the higher the photocurrent densities were, regardless of the applied potential. This fact was related to an increase in the average diameter of the initiation layer porosity and/or the removal of this layer when anodization was performed when the electrode was stirred, which enhanced solar light absorption. Second, regardless of the hydrodynamic conditions used for the synthesis of the photocatalysts, the external anodic potential over the nanostructures increased the space charge layer and band bending, thus suppressing the recombination of photogenerated electron/hole pairs [8]. For this reason, as the applied potential increased, the photocurrent response was higher: However, from −0.25 V_Ag/AgCl_ the photocurrent stabilized. This indicated that, from this potential value, the photocatalyst was saturated, and a higher potential did not enhance the space charge layer or the band bending. As the principal concern in a reaction promoted by light is to account for the numbers of photons driving [21], incident photon-to-current efficiency (IPCE) measurements were performed on the samples anodized at *Re* = 0 and *Re* = 600 (Figure S3). IPCE describes the photocurrent collected per incident photon flux as a function of the illumination wavelength [22]. IPCE measurements were performed in 0.1 M Na_2_SO_4_ under an applied potential of 0.5 V (vs Ag/AgCl) and in a wavelength region of 300 to 500 nm. The IPCE was calculated as follows:

(2) $$\mathrm{IPCE}=\frac{1240\cdot i}{P\cdot \lambda }\cdot 100,$$

where i is the photocurrent density expressed in A·cm^−2^, *P* is the light power density in W·cm^−2^, and λ is the wavelength in nm. As expected, the IPCE values of the nanostructures synthetized at *Re* = 600 were higher than the ones obtained under static conditions (Figure S3).

### 3.2. Reaction Kinetics

Acetaminophen has a maximum absorption band at 243 nm associated with the C=C bonds of the aromatic ring [23]. Several works that have studied the degradation of acetaminophen have used this band in order to evaluate its removal over time, since this peak gradually decreases as the degradation is carried out [4,23]. According to this, it is assumed that the concentration of acetaminophen is proportional to the absorbed radiation in the spectrophotometer (the Beer–Lambert law) [24]. Hence, the degradation of acetaminophen might be evaluated from UV-visible spectra due to the relationship between concentration and absorbance. 

On the other hand, the Langmuir–Hinshelwood (L–H) model has been successfully applied to explain the photocatalytic reaction kinetics for aquatic organics [25]. In particular, when the concentration of the organic pollutant is low (0.1–0.5 mM~15–75 mg·L^−1^ [24]), the L–H model is expressed as a pseudo-first-order model [26]:

(3) $$r\left(t\right)=\frac{k_{r}\cdot k_{ad}\cdot C\left(t\right)}{1+k_{ad}\cdot C\left(t\right)}\approx k_{r}\cdot k_{ad}\cdot C\left(t\right)=k_{app}\cdot C\left(t\right),$$

where $r\left(t\right)$ is the degradation rate at time *t*; $k_{r}$ is the intrinsic rate constant; $k_{ad}$ is the adsorption equilibrium constant; $k_{app}$ is the pseudo-first-order rate constant of the degradation of acetaminophen during photoelectrocatalysis; and *C(t)* is the concentration of AMP at time *t*.

In addition, the photoelectrochemical cell is considered to be an ideal batch reactor. Thus, it is possible to express the mass balance in this cell according to Equation (4):

(4) $$\frac{dC\left(t\right)}{dt}=-r\left(t\right)=\;-k_{app}\cdot C\left(t\right).$$

Finally, Equation (3) and Equation (4) can be rearranged as

(5) $$\int_{C_{0}}^{C\left(t\right)}\frac{dC\left(t\right)}{C\left(t\right)}=-k\int_{0}^{t}dt=\ln\left(\frac{C\left(t\right)}{C_{0}}\right)=-k_{app}\cdot t,$$

where $C_{0}$ is the initial concentration of AMP. 

To sum up, Equation (5) is obtained assuming that (1) photoelectrocatalysis kinetics follow the L–H model, (2), the concentration of acetaminophen in the photoelectrochemical cell is low (40 mg·L^−1^ in this study), and (3) the photoelectrochemical cell behaves as an ideal batch reactor.

### 3.3. Electrocatalysis, Photocatalysis, and Photoelectrocatalysis

**Figure 2:**
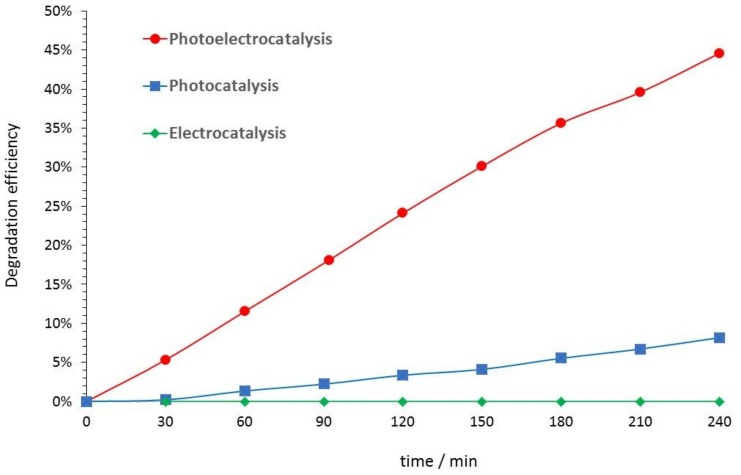
Monitoring of the photoelectrocatalytic, photocatalytic, and electrocatalytic degradation of acetaminophen by means of absorbance measurements with an ultraviolet-visible spectrophotometer. The photocatalyst was anodized at a Re of 600 (length = 6.1 µm), and the pH value was 7.

Figure 2 shows the differences between the electrocatalysis, photocatalysis, and photoelectrocatalysis processes for acetaminophen degradation. The TiO_2_ photocatalyst was anodized at a Re of 600, and the pH used was 7. According to Figure 2, degradation was not possible when only an external anodic potential was applied (electrocatalysis), since there was no driving force (electromagnetic radiation) that allowed for the generation of electron/hole pairs. 

Otherwise, if only electromagnetic radiation was used (photocatalysis), electron/hole pairs were generated, but their recombination prevailed over kinetic photodegradation, and therefore a low degradation was obtained over time.

However, in the photoelectrocatalysis process, a synergistic effect was observed between both factors. That is, the anodic potential avoided part of the recombination of electron/hole pairs, which had been previously formed with the interaction between the electromagnet radiation and the catalyst surface. 

### 3.4. Effect of the Reynolds Number and pH on Acetaminophen Degradation

**Figure 3:**
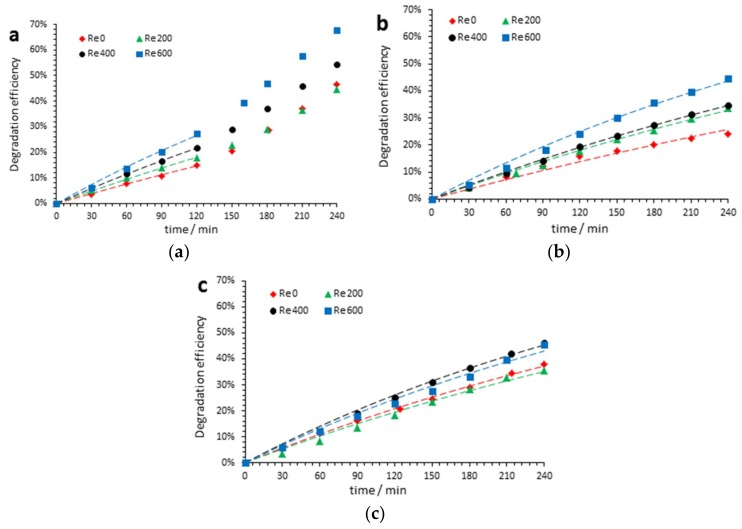
Photoelectrocatalytic (PEC) degradation efficiencies of acetaminophen by means of absorbance measurements with an ultraviolet-visible spectrophotometer with TiO_2_ nanostructures anodized (at Re 0, 200, 400, and 600 with lengths of 3.7, 5.0, 5.4, and 6.1 µm, respectively) during the 240 min of the degradation test for (**a**) pH 3, (**b**) pH 7, and (**c**) pH 9.

Figure 3 shows that the PEC degradation of acetaminophen depended on the hydrodynamic conditions used during anodization of the samples. Table 1 shows $X_{AMP}$ $\left(\frac{C_{0}-C\left(t\right)}{C_{0}}=\;\frac{A_{0}-A\left(t\right)}{A_{0}}\right)$, where $A_{0}$ is the initial absorbance at 243 nm, and $A\left(t\right)$ is the absorbance at 243 nm and time *t* at the end of the experiment ($X_{AMP_{240}}$), $k_{app}$, and the coefficient of determination (*R*^2^). The latter was calculated through an ordinary least square regression, taking into account the model expressed in Equation (5).

In order to study the influence of each factor on the degradation rate, a regression model was proposed. This model takes into account the relationship between $X_{AMP_{240}}$ and the lineal effects of Re ($\beta_{1}$) and pH ($\beta_{2}$), the quadratic effects of Re ($\beta_{3}$) and pH ($\beta_{4}$), and finally the effects of their interaction ($\beta_{5}$). This model is given as follows:

(6) $$E\left(X_{AMP_{240}}\right)=\beta_{0}+\beta_{1}\cdot Re+\beta_{2}\cdot pH+\beta_{3}\cdot Re^{2}+\beta_{4}\cdot pH^{2}+\beta_{5}\cdot Re\;\times pH,$$

where $E\left(X_{AMP_{240}}\right)$ is the expected value of $X_{AMP_{240}}$, and $\beta_{0}$ is the expected value of $X_{AMP_{240}}$ when Re and pH are 0. Note that there is no physical meaning for $\beta_{0}$.

Additionally, it was crucial to analyze the results from a statistical point of view, in order to know which effects are really statistically significant and which are not.

For that reason, Fisher’s test based on an analysis of variance (ANOVA) was carried out (Table 2), where the sum of the squares represents a measure of variation or deviation with respect to the average for each factor effect and the residual error, and where the *p*-value for each effect states the probability of obtaining the same results when the null hypothesis is correct (i.e., that the effect is not significant). In this work, it was assumed that when the *p*-value was less than 0.05, the effect was significant, because there was not enough statistical evidence to accept the null hypothesis. It is important to point out that the quadratic effect of Re ($\beta_{3}$) and the interaction between Re and pH ($\beta_{5}$) are not shown in Table 2, because a previous ANOVA analysis determined that these effects were not significant.

Figure 3 and Table 1 show that, in general and regardless of the pH, the degradation rate increased as the hydrodynamic conditions applied during anodization also increased. This fact could have been related to the total or partial removal of the initiation layer when the electrode was stirred during anodization. Table 2 confirms this idea, since there was enough statistical evidence to determine that the estimated lineal effect of Re was significant and positive. 

On the other hand, Figure 3 and Table 1 also show that the results corresponding to pH 7 and 9 perfectly fit the model expressed in Equation (5) (high *R*^2^): Nevertheless, when PEC degradation was performed at pH 3, the situation changed (that is, the data obtained at pH 3 only fit Equation (5) during the first 120 min of the degradation test) (see Figure 3a and Table 1). 

Some studies have stated that as the pH increases, a higher number of hydroxide groups are available on the TiO_2_ surface. Therefore, they can be easily oxidized and form a hydroxyl radical, which favors the degradation of acetaminophen [6]. However, at pH values higher than the pKa of the drug (9.5 for acetaminophen), it tends to exist in an anion form. Therefore, when the pH is gradually increased from 9.5, the electrostatic repulsion forces between the TiO_2_ surface (pH_PZC_: pH at the isoelectric point 6.3) and acetaminophen are also increased, and thus the expected degradation rate decreases [6]. 

All of these facts are consistent with the results shown in Table 1 for pH 7 and 9, since the PEC degradation of acetaminophen over time increased from pH 7 to 9 regardless of the hydrodynamic conditions. However, maximum PEC degradation was obtained at pH 3. This could have been associated with the chloride ions present in the solution from the dissociation of HCl used to adjust the pH. Then, the anodic potential applied conducted to an electro-oxidation to the ${\mathrm{Cl}}^{-}$ and generated soluble chlorine (${\mathrm{Cl}}_{2}\left(\mathrm{aq}\right)$) according to Equation (7) [16]:

(7) $$2{\mathrm{Cl}}^{-}\to {\mathrm{Cl}}_{2}\left(\mathrm{aq}\right)+2e^{-}.$$

In this case, the potential needed for Cl_2_ formation was 1.36 V_NHE_, and the holes formed in the valence band of TiO_2_ had a potential of 2.70 V_NHE_ [27]: Thus, Cl_2_ could be generated.

Chlorine was subsequently hydrolyzed and transformed into chloride ion and hypochlorous acid (HClO) Equation (8). The latter (pKa_HClO_ = 7.55) was in equilibrium with the hypochlorite ion (${\mathrm{ClO}}^{-}$) (Equation (9)) [16]:

(8) $$Cl_{2}\left(aq\right)+H_{2}O\underset{eq}{\Leftrightarrow }HClO+Cl^{-}+H^{+},$$

(9) $$HClO\underset{eq}{\Leftrightarrow }ClO^{-}+H^{+}.$$

At equilibrium, Boxall and Kelsall found out that ${\mathrm{Cl}}_{2}$ is predominant until a pH close to 3, HClO in the pH range of 3–8, and ${\mathrm{ClO}}^{-}$ at a pH higher than 8 [28]. Therefore, when PEC degradation is performed at pH 3, ${\mathrm{Cl}}_{2}$ and HClO species are generated, and they react with the acetaminophen in competition with the degradation based on the oxidation by hydroxyl radicals.

According to Figure 3 and Figure S4, when PEC degradation took place at pH 3, the oxidation driving force came from the hydroxyl radicals during the first 120 min, and therefore the degradation showed a pseudo-first-order behavior (Table 1, bottom of the table). However, from this moment, this behavior changed due to the predominant attack of ${\mathrm{Cl}}_{2}$ and HClO species. The increase in the PEC degradation could be attributed to the reactivity of HClO. In fact, at pH 3, degradation of acetaminophen continued even when the PEC reaction had finished. Table 2 shows a positive quadratic significant effect of pH, as explained before.

**Figure 4:**
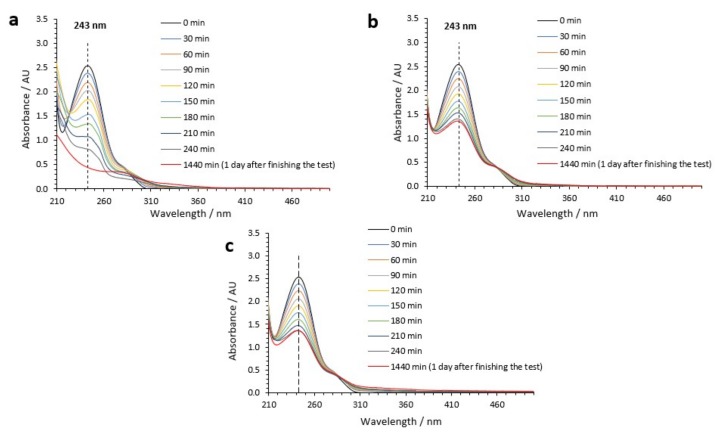
UV-visible absorption spectra obtained during PEC degradation of acetaminophen with TiO_2_ photocatalysts anodized at *Re* = 600 (length = 6.1 µm) for (**a**) pH 3, (**b**) pH 7, and (**c**) pH 9.

Figure 4 shows the UV-visible spectra at different times during the PEC reaction for the nanostructures anodized at *Re* = 600. Figure 4b,c presents a similar trend for the evolution of the UV-visible spectra with time. This was related to the pseudo-first-order mechanism followed when acetaminophen PEC degradation was carried out at pH 7 and 9. However, Figure 4a shows that from 120 min the trend of the spectra was completely different due to the attack of species formed at pH 3 (${\mathrm{Cl}}_{2}$ and HClO). To sum up, it could be stated that at pH = 9, a higher number of hydroxyl radicals enhanced acetaminophen photodegradation in comparison to pH = 7. However, at pH = 3, a different mechanism involved in the process promoted the generation of Cl_2_ and HClO, increasing the degradation rate.

### 3.5. Study of the Byproducts 

The oxidation of acetaminophen by means of either hydroxyl radicals or electro-oxidation in the presence of HCl leads to the generation of different byproducts according to each mechanism, which might introduce a higher environmental risk. Some studies performed with high-performance liquid chromatography (HPLC) have observed that there are three main categories of generated by-products during acetaminophen photodegradation: Aromatic compounds, carboxylic acids, and inorganic compounds [29,30].

When degradation is carried out by hydroxyl radicals, hydroquinone is usually the main detected intermediate due to a hydroxyl radical attack on the aromatic ring of acetaminophen [31]. Thus, a para-substitution is carried out. Hydroquinone presented a characteristic absorption band in the UV-visible spectrophotometry spectra at a wavelength of 290 nm. Figure 4 shows an absorbance peak at 290 nm, which increased with PEC reaction time. This peak was associated with the characteristic absorption band of hydroquinone. However, when PEC photodegradation was carried out at pH 3, this peak started to decrease from 120 min, and absorbance peaks at higher wavelengths appeared. In particular, characteristic absorption bands at 300 nm and 320 nm could be elucidated (Figure 4a) and were related to the formation of p-aminophenol and p-nitrophenol, respectively [32,33]. This confirmed the different mechanism of PEC degradation in the presence of HCl. 

According to all of these facts, a higher efficiency of acetaminophen PEC degradation was obtained at pH 3. However, a higher diversity of byproducts was generated, which could be a drawback if these compounds are not completely removed.

### 3.6. COD and TOC Tests

Table 3 shows the results of the COD efficiencies for all the samples at the end of the PEC degradation (Table 3) and TOC efficiencies (Table 3) for the nanostructures anodized at *Re* = 600. From Table 3, it can be deduced that, in general, the COD efficiency values increased as the hydrodynamic conditions applied during the anodization also increased. Moreover, the highest COD efficiencies were obtained when PEC degradation was carried out at pH 3: However, it was not possible to observe significant differences between the COD efficiency values obtained at pH 7 and 9. Additionally, Table 3 shows that the highest state of mineralization was obtained at pH 3. 

Therefore, COD and TOC tests are consistent with the obtained results.

## 4. Conclusions

This work studied the influence of pH on the PEC degradation of acetaminophen with TiO_2_ nanotubes anodized under different hydrodynamic conditions.

As the Reynolds number increased, the initiation layer that totally or partially covered the TiO_2_ nanotubes disappeared. As a consequence of this, PEC degradation efficiency linearly increased with the Reynolds number, regardless of the pH used during the degradation. Statistically significant analysis confirmed this behavior.

In relation to the pH influence, for pH 7 and 9, the degradation mechanism was based on the oxidation by hydroxyl radicals, with a pseudo-first-order kinetic behavior. Under these conditions, when the pH (from 7 to 9) increased, the formation of ·OH also increased, and therefore a higher PEC degradation of acetaminophen was observed. However, for pH 3, a more reactive mechanism was initiated after 120 min of PEC reaction due to the predominant attack of ${\mathrm{Cl}}_{2}$ and HClO species. This reactive mechanism caused a higher degradation at the end of the experiment and also a higher number of generated byproducts during the PEC degradation. Finally, COD and TOC tests also showed that for pH 3, PEC degradation was faster and a higher degree of mineralization state was achieved.

## Figures and Tables

**Table 1:** Values of the kinetic parameters according to pseudo-first-order kinetics and the coefficient of determination (*R*^2^) for the data fitted for 240 min of reaction time (top of the table) and for data fitted for the first 120 min of reaction time (bottom of the table).

	***Re***	***k_app_* (h^−1^)**	*R* ^2^	$X_{AMP}{}_{240}$ (%)
**pH 3**	0	0.1234	0.8622	46.47
	200	0.1250	0.9272	44.58
	400	0.1655	0.9179	38.66
	600	0.2299	0.8906	67.73
**pH 7**	0	0.0747	0.9685	24.18
	200	0.0998	0.9945	33.61
	400	0.1064	0.9984	34.79
	600	0.1438	0.9934	44.58
**pH 9**	0	0.1166	0.9969	37.83
	200	0.1084	0.9888	35.54
	400	0.1512	0.9938	46.19
	600	0.1407	0.9824	45.40
	***Re***	***k_app_* (h^−1^)**	**R^2^**	
**pH 3**	0	0.0797	0.9948	
	200	0.0999	0.9961	
	400	0.1223	0.9993	
	600	0.1547	0.9872	

**Table 2:** Test F based on an analysis of variance of the efficiency degradation of the Reynolds, pH, and quadratic pH effects.

	Estimated β	Sum of Squares	Gl	Mean Square	*F_ratio_*	*p*-Value
**Re (β_1_)**	15.36	393.22	1	393.22	8.18	**0.0212**
**pH (β_2_)**	−8.12	131.87	1	131.87	2.74	0.1363
**pH^2^ (β_4_)**	22.02	323.25	1	323.25	6.72	**0.0320**
**Residual error**		384.67	8	48.08		
**Total**		1233.00	11			

**Table 3:** Chemical oxygen demand (COD) efficiency values for the nanostructures anodized at the different Re values and for the different pH values and total organic carbon (TOC) efficiency values for the nanostructures anodized at *Re* = 600 and at the different pH values.

	***Re***	**(COD_0_ − COD)/COD_0_**
**pH 3**	0	0.30
	200	0.45
	400	0.66
	600	0.60
**pH 7**	0	0.27
	200	0.37
	400	0.32
	600	0.35
**pH 9**	0	0.21
	200	0.13
	400	0.32
	600	0.49
	***Re***	**(TOC_0_ − TOC)/TOC_0_**
**pH 3**	600	0.45
**pH 7**		0.21
**pH 9**		0.24

## Supplementary Materials

The following are available online at https://www.mdpi.com/2079-4991/9/4/583/s1, Figure S1. Structure of acetaminophen, Figure S2. (a) Scheme of the electrode configuration during electrochemical anodization. (b) Photograph of the photoelectrochemical setup for electrochemical characterization of the TiO2 samples: 1. Working electrode, 2. Reference electrode and 3. Counter electrode, IPCE measurements for the nanotubes anodized at Re = 0 (length = 3.7 μm) and Re = 600 (length = 6.1 μm), Figure S4. Pseudo-first order kinetics obtained from the fitting of the PEC-degradation efficiencies of acetaminophen (Figure 3) with TiO2 nanostructures anodized (at Re 0, 200 400 and 600 with lengths of 3.7, 5.0, 5.4 and 6.1 μm, respectively) for (a) pH 3, (b) pH 7 and (c) pH 9.

## References

[1] Rivera-Utrilla J., Sánchez-Polo M., Ferro-García M.A., Prados-Joya G., Ocampo-Perez R. (2013). Pharmaceuticals as emerging contaminants and their removal from water. A review. Chemosphere.

[2] Yang L., Yu L.E., Ray M.B. (2008). Degradation of paracetamol in aqueous solutions by TiO_2_ photocatalysis. Water Res..

[3] Solís-Casados D., Escobar-Alarcón L., Gómez-Oliván L., Haro-Poniatowski E., Klimova T. (2017). Photodegradation of pharmaceutical drugs using Sn-modified TiO_2_ powders under visible light irradiation. Fuel.

[4] Xie G., Chang X., Adhikari B.R., Thind S.S., Chen A. (2016). Photoelectrochemical degradation of acetaminophen and valacyclovir using nanoporous titanium dioxide. Chin. J. Catal..

[5] Basha S., Keane D., Nolan K., Oelgemöller M., Lawler J., Tobin J.M., Morrissey A. (2015). UV-induced photocatalytic degradation of aqueous acetaminophen: The role of adsorption and reaction kinetics. Environ. Sci. Pollut. Res..

[6] Jallouli N., Elghniji K., Trabelsi H., Ksibi M. (2017). Photocatalytic degradation of paracetamol on TiO_2_ nanoparticles and TiO_2_/cellulosic fiber under UV and sunlight irradiation. Arab. J. Chem..

[7] El-Kemary M., El-Shamy H., El-Mehasseb I. (2010). Photocatalytic degradation of ciprofloxacin drug in water using ZnO nanoparticles. J. Lumin..

[8] Lin C.J., Liao S.-J., Kao L.-C., Liou S.Y.H. (2015). Photoelectrocatalytic activity of a hydrothermally grown branched ZnO nanorod-array electrode for paracetamol degradation. J. Hazard. Mater..

[9] Eftekhari A., Babu V.J., Ramakrishna S. (2017). Photoelectrode nanomaterials for photoelectrochemical water splitting. Int. J. Hydrogen Energy.

[10] Sánchez-Tovar R., Fernández-Domene R.M., García-García D.M., García-Antón J. (2015). Enhancement of photoelectrochemical activity for water splitting by controlling hydrodynamic conditions on titanium anodization. J. Power Sources.

[11] Roy P., Berger S., Schmuki P. (2011). TiO_2_ Nanotubes: Synthesis and Applications. Angew. Chem. Int. Ed..

[12] Diebold U. (2003). The surface science of titanium dioxide. Surf. Sci. Rep..

[13] Mills A., le Hunte S. (1997). An overview of semiconductor photocatalysis. J. Photochem. Photobiol. A.

[14] Tovar R.S., Paramasivam I., Lee K., Schmuki P. (2012). Influence of hydrodynamic conditions on growth and geometry of anodic TiO_2_ nanotubes and their use towards optimized DSSCs. J. Mater. Chem..

[15] Borràs-Ferrís J., Sánchez-Tovar R., Blasco-Tamarit E., Fernández-Domene R.M., García-Antón J. (2016). Effect of Reynolds number and lithium cation insertion on titanium anodization. Electrochim. Acta.

[16] Zavala M.A.L., Estrada E.E. (2016). Degradation of Acetaminophen and Its Transformation Products in Aqueous Solutions by Using an Electrochemical Oxidation Cell with Stainless Steel Electrodes. Water.

[17] (2012). Standard Methods for the Examination of Water and Wastewater.

[18] Costa L.L., Prado A.G. (2009). TiO_2_ nanotubes as recyclable catalyst for efficient photocatalytic degradation of indigo carmine dye. J. Photochem. Photobiol. A Chem..

[19] Hsiao P.-T., Wang K.-P., Cheng C.-W., Teng H. (2007). Nanocrystalline anatase TiO_2_ derived from a titanate-directed route for dye-sensitized solar cells. J. Photochem. Photobiol. A Chem..

[20] Qian L., Du Z.-L., Yang S.-Y., Jin Z.-S. (2005). Raman study of titania nanotube by soft chemical process. J. Mol. Struct..

[21] Hoque M.A., Guzman M.I. (2018). Photocatalytic Activity: Experimental Features to Report in Heterogeneous Photocatalysis. Materials.

[22] Chen Z., Jaramillo T.F., Deutsch T.G., Kleiman-Schwarsctein A., Forman A.J., Gaillard N., Garland R., Takanabe K., Heske C., Sunkara M. (2010). Accelerating Materials Development for Photoelectrochemical Hydrogen Production: Standards for Methods, Definitions, and Reporting Protocols. J. Mater. Res..

[23] Aguilar C.A., Montalvo C., Ceron J.G., Moctezuma E. (2011). Photocatalytic Degradation of Acetaminophen. Int. J. Environ. Res..

[24] Chang S.L., Fekete M., Hocking R.K., Izgorodina A., Singh A., Zhou F., Macfarlane D.R., Spiccia L. (2013). Role of Advanced Analytical Techniques in the Design and Characterization of Improved Catalysts for Water Oxidation. New and Future Developments in Catalysis: Solar Photocatalysis.

[25] Lin J.C.-T., De Luna M.D.G., Aranzamendez G.L., Lu M.-C. (2016). Degradations of acetaminophen via a K_2_S_2_O_8_-doped TiO_2_ photocatalyst under visible light irradiation. Chemosphere.

[26] Malakootian M., Pourshaban-Mazandarani M., Hossaini H., Ehrampoush M.H. (2016). Preparation and characterization of TiO_2_ incorporated 13X molecular sieves for photocatalytic removal of acetaminophen from aqueous solutions. Process Saf. Environ. Prot..

[27] Paramasivam I., Jha H., Liu N., Schmuki P. (2012). A Review of Photocatalysis using Self-organized TiO_2_ Nanotubes and Other Ordered Oxide Nanostructures. Small.

[28] Boxall C., Kelsall G.H. (1992). Hypochlorite Electrogeneration. II. Thermodynamics and Kinetic Model of the Anode Reaction Layer.

[29] Yang L., Yu L.E., Ray M.B. (2009). Photocatalytic Oxidation of Paracetamol: Dominant Reactants, Intermediates, and Reaction Mechanisms. Environ. Sci. Technol..

[30] Tao H., Liang X., Zhang Q., Chang C.-T. (2015). Enhanced photoactivity of graphene/titanium dioxide nanotubes for removal of Acetaminophen. Appl. Surf. Sci..

[31] Zhang X., Wu F., Wu X., Chen P., Deng N. (2008). Photodegradation of acetaminophen in TiO_2_ suspended solution. J. Hazard. Mater..

[32] Çifçi D.I., Tunçal T., Pala A., Uslu O. (2016). Determination of optimum extinction wavelength for paracetamol removal through energy efficient thin film reactor. J. Photochem. Photobiol. A Chem..

[33] Moctezuma E., Leyva E., Aguilar C.A., Luna R.A., Montalvo C. (2012). Photocatalytic degradation of paracetamol: Intermediates and total reaction mechanism. J. Hazard. Mater..

