# Supplementary material for: TiO2 Nanostructures for Photoelectrocatalytic Degradation of Acetaminophen
Source: Nanomaterials (Basel). 2019 Apr 9;9(4):583. doi: 10.3390/nano9040583 (PMC6523489; doi:10.3390/nano9040583)
Supplement: Supplementary file 1 [file nanomaterials-09-00583-s001.pdf]

nanomaterials

MDPI

Supplementary Materials

TiO_2 Nanostructures for Photoelectrocatalytic Degradation of Acetaminophen

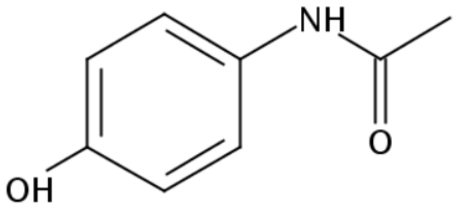

Figure S1. Structure of acetaminophen.

a)

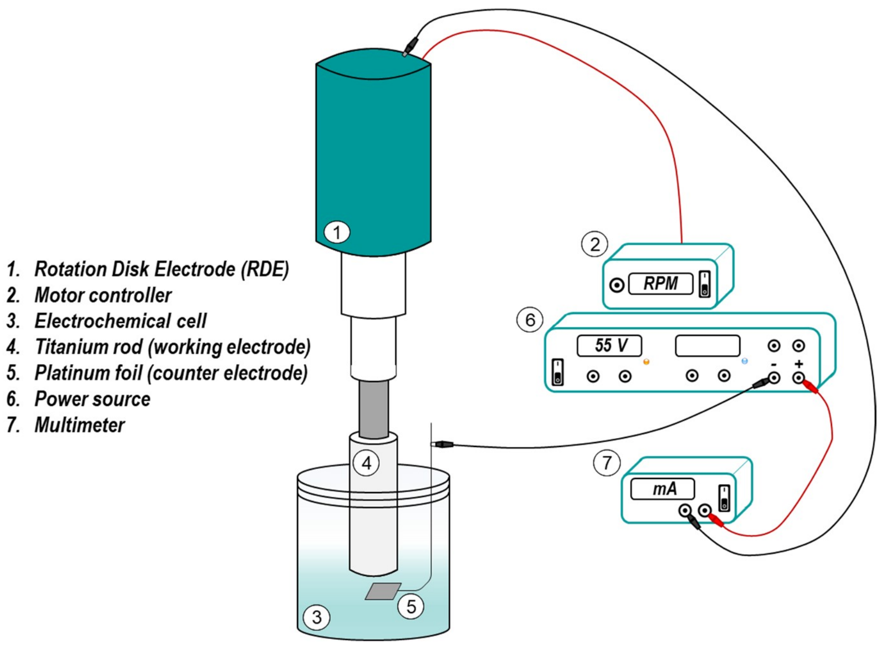

b)

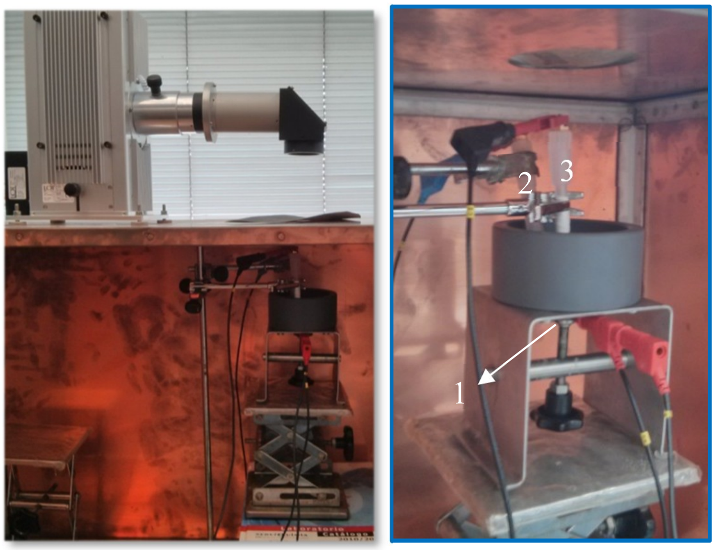

Figure S2. **(a)** Scheme of the electrode configuration during electrochemical anodization. **(b)** Photograph of the photoelectrochemical setup for electrochemical characterization of the TiO_2 samples: (1) working electrode, (2) reference electrode, and (3) counter electrode.

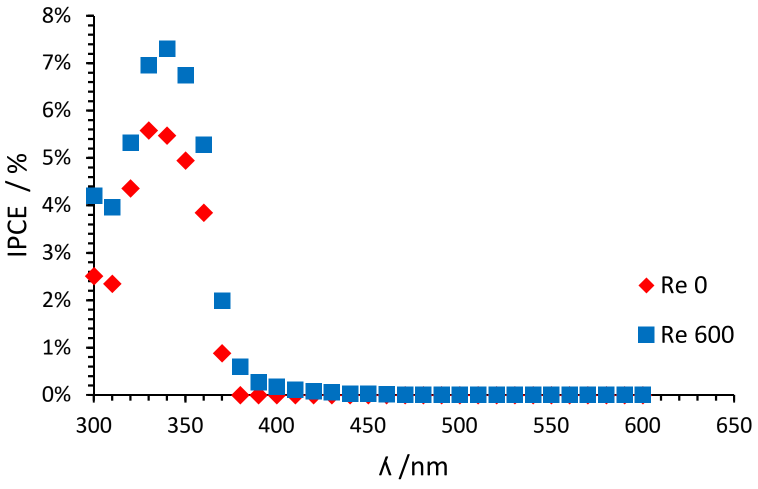

Figure S3. Incident photon-to-current efficiency (IPCE) measurements for the nanotubes anodized at *Re* = 0 (length = 3.7 μm) and *Re* = 600 (length = 6.1 μm).

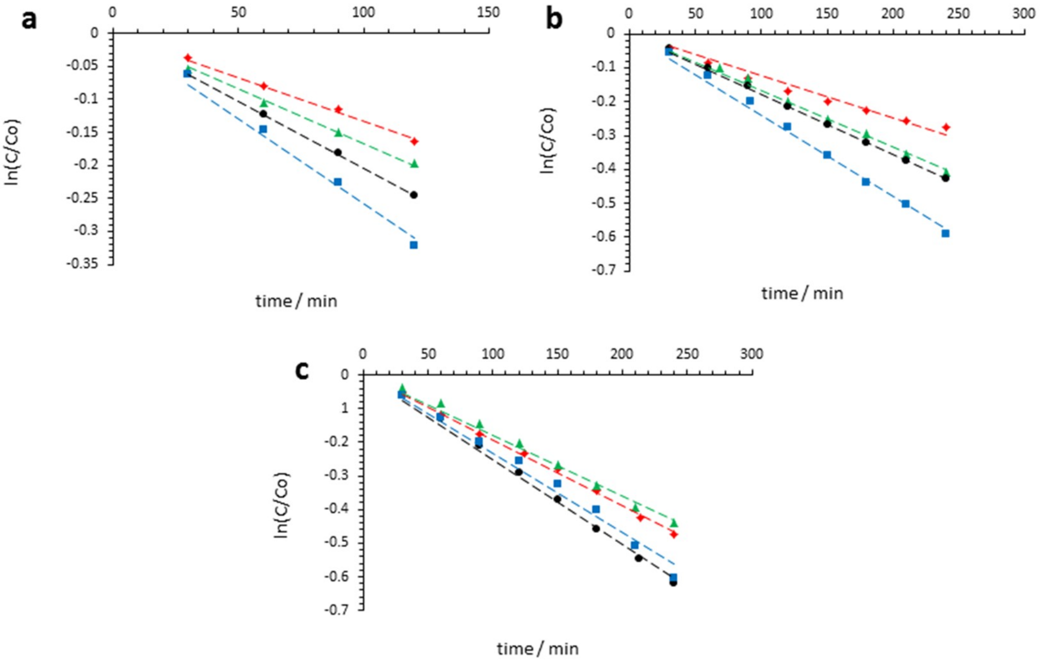

Figure S4. Pseudo-first-order kinetics obtained from the fitting of the PEC degradation efficiencies of acetaminophen (Figure 3) with TiO_2 nanostructures anodized (at *Re* 0, 200, 400, and 600 with lengths of 3.7, 5.0, 5.4, and 6.1 µm, respectively) for **(a)** pH 3, **(b)** pH 7, and **(c)** pH 9.
